# The Role of the Autoimmune Regulator Gene in the Control of MHC II Antigen‐Processing and Presentation by Medullary Thymic Epithelial Cells

**DOI:** 10.1111/tan.70516

**Published:** 2025-12-22

**Authors:** A. C. Monteleone‐Cassiano, R. D. Pinto, P. Ferreirinha, U. G. Cipriano, A. F. Gembre, V. L. D. Bonato, G. A. Passos, N. L. Alves, E. A. Donadi

**Affiliations:** ^1^ Basic and Applied Immunology Program, Ribeirão Preto Medical School University of São Paulo Ribeirão Preto São Paulo Brazil; ^2^ Instituto de Investigação e Inovação em Saúde (i3S), University of Porto Porto Portugal; ^3^ Department of Biochemistry and Immunology, Ribeirao Preto Medical School University of Sao Paulo São Paulo São Paulo Brazil; ^4^ Department of Basic and Oral Biology, Ribeirão Preto School of Dentistry University of São Paulo Ribeirão Preto São Paulo Brazil; ^5^ Department of Medicine Ribeirão Preto Medical School, Division of Clinical Immunology University of São Paulo Ribeirão Preto São Paulo Brazil

**Keywords:** *Aire* gene, antigen presentation, antigen processing, MHC II molecules, mTEC

## Abstract

The autoimmune regulator (*AIRE*) gene deficiency in humans causes the autoimmune polyglandular syndrome type 1 (APS‐1). *AIRE* contributes to self‐tolerance and prevents autoimmunity via the regulation of tissue‐restricted antigen (TRA) expression in medullary thymic epithelial cells (mTECs). Using a murine *Aire*‐deficient mTEC line (*Aire* ^−/−^), we previously reported that *Aire* regulates cell adhesion genes, affecting homotypic mTEC/mTEC and heterotypic mTEC/thymocyte interaction in vitro. Transcriptional analysis comparing *Aire* ^*WT*^ with *Aire* ^*−/−*^ mTECs indicates that *Aire* modulates antigen processing and presentation genes. *Aire* ^−/−^ mTECs, but not *Aire* ^*WT*^, exhibited delayed expression of MHC II and CD74 molecules, suggesting that *Aire* influences the MHC II presentation pathway. However, *Aire* ^*WT*^ and *Aire* ^−/−^ mTECs did not directly present the OVA^323–339^ peptide to OT‐II TCR transgenic single‐positive CD4+ T cells. When cocultured with splenocytes and dendritic cells, both mTEC types induce T cell activation and present the OVA^323–339^ peptide to OT‐II TCR transgenic T cells. To further understand *Aire'*s role in MHC II antigen processing/presentation, we evaluated the expression of the MHC II transactivator (*Ciita*), IFN‐γ receptors and IFN‐γ‐induced GTPases, as well as lysosome and cathepsin L activities. After IFN‐γ treatment, *Aire* ^−/−^ mTECs, but not *Aire* ^*WT*^, delayed the expression of *Ciita*. Our results suggest *that Aire* controls some steps of the MHC II antigen processing/presentation‐related proteins.

## 1 Introduction

The cortical and medullary thymus compartments promote the development and selection of immunologically competent and self‐tolerant T cells. Cortical thymic epithelial cells (cTECs) drive lineage commitment and positive selection and the medullary thymic epithelial cells (mTECs) control the negative selection of potential autoreactive T cells or their differentiation into T regulatory cells (Tregs) [1, 2]. mTECs can ectopically express nearly all protein‐coding genes, encompassing thousands of tissue‐restricted antigens (TRAs). This diverse gene expression in the thymic medulla, also known as promiscuous gene expression (pGE), is partially regulated by the autoimmune regulator (*AIRE* in humans and *Aire* in mice) gene, contributing to self‐immunological tolerance [3, 4].

The human AIRE protein has 80% identity to the murine Aire protein, exhibiting five significant domains: (i) the caspase recruiter domain (CARD), encoded by exons 1 and 2; (ii) the nuclear location signal (NLS) domain, encoded by exon 3; (iii) the SAND (Sp100, AIRE‐1, NucP41/75, DEAF‐1) domain, encoded by exons 5–7 and (iv) two zinc finger type plant homeodomains (PHD‐1 encoded by exons 8 and 9 and PHD‐2 encoded by exons 11 and 12). Each AIRE domain presents specific functions: (i) CARD is involved in the oligomerisation of AIRE, (ii) the NLS domain is responsible for transporting the protein from the cytoplasm to the nucleus, (iii) the SAND domain promotes DNA binding and (iv) the two PHDs function as histone code readers [5, 6, 7]. Reduced expression of TRAs in *Aire*‐deficient mTECs impairs the elimination of autoreactive T cells and the production of Tregs [8]. pGE ensures that developing T cells encounter self‐antigens during their maturation process, eliminating potentially harmful self‐reactive T cells or deviating them to the Treg lineage, preventing autoimmune disorders [9, 10].

Mutations in the *AIRE* gene have been associated with a rare inherited autoimmune disorder denominated autoimmune polyendocrine syndrome type 1 (APS‐1), also known as APECED (autoimmune polyendocrinopathy candidiasis ectodermal dystrophy). APS‐1 patients present autoimmune manifestations in multiple organs, including hypoparathyroidism and primary adrenocortical failure, associated with chronic mucocutaneous candidiasis [11, 12, 13]. To promote the expression of TRAs, *AIRE* induces the transcription of ectopically autoantigens in the thymus. mTECs may directly perform self‐antigen presentation to developing thymocytes in the context of MHC molecules [14, 15, 16]. In addition, thymic dendritic cells (DCs) can acquire antigens via cooperative antigen transfer from mTECs [17] and indirectly present them to CD4^+^ and CD8^+^ T cells [18]. Therefore, several layers of antigen presentation within the thymus are critical to prevent the escape of autoreactive T cells, to promote immunological tolerance and to avoid aggressive autoimmunity [19, 20].

The functional relationship between *Aire* and the antigen processing and presentation by MHC II molecules has not been completely elucidated. Different mouse models revealed that the absence or dysfunction of the SAND, CARD and PHD1 Aire protein domains modifies the expression of MHC II molecules [21, 22, 23]. In particular, a thymic epithelial cell line (mTEC 3.10), established from a newborn C57BL/6 mouse, exhibits a typical mTEC morphology and phenotype [24, 25, 26]. We have used the mTEC 3.10 line as a model system to evaluate (i) the mTEC transcriptional profile [27, 28], (ii) the expression profile of TRA genes [29, 30], (iii) the thymocyte interaction with mTECs [31], (iv) the mTEC‐mTEC adhesion [32], (iv) the long noncoding RNA (lncRNA) profile [33] and (v) the network of interactions between various signalling molecules and transcription factors that regulate immune responses [34]. In this study, we employed *Aire* ^*WT*^ and an *Aire* ^−/−^ murine mTEC lines [35] to study the role of *Aire* in the orchestration of MHC II‐antigen processing and presentation by mTECs. We found that *Aire* controls the IFN‐γ‐induction of MHC II, CD74 and Ciita expression, suggesting a novel contribution for *Aire* in mTEC biology and function.

## 2 Materials and Methods

### 2.1 mTEC Lines

We used the *Mus musculus* wild‐type (WT) (*Aire* ^*WT*^) mTEC 3.10 cell line [24, 31, 36] and the *Aire*‐deficient mTEC 3.10 E6 cell clone (E6), which was obtained through the CRISPR‐Cas9 system [35]. The mTEC 3.10 E6 clone carried two indel mutations, affecting both *Aire* alleles (compound heterozygous), hereafter referred to as *Aire* ^*−/−*^. The *Aire* allele 1 carries two types of mutations: T>G substitution (mRNA nucleotide position 351), followed by a nine‐base pair deletion (GCTGGTCCC, mRNA, positions 352–360) that transcribed a 1647‐nucleotide *Aire* mRNA. The allele 2 carries a single G deletion at mRNA at position 352, transcribing a 1,655‐nucleotide *Aire* mRNA. Both mTEC 3.10 E6 alleles produced nonfunctional Aire proteins, so this cell was considered *Aire* knockout, that is, *Aire* ^−/−^.

### 2.2 Flow Cytometry Analyses

To evaluate the thymic medullary phenotype of *Aire* ^*WT*^ and *Aire* ^−/−^ after IFN‐γ or LPS stimulation, the cell suspensions were stained with anti‐mouse MHC II‐A780, anti‐mouse MHC I (H2Kb)‐PE (BD Biosciences, San Jose, CA) and anti‐mouse CD74‐A647 (intracellular staining using the Intracellular Fixation & Permeabilization Buffer Set, Thermo‐Fisher, Waltham, MA). Flow cytometry was performed in all experiments using a BD LSRFortessa Cell Analyser (Becton Dickinson) and data were analysed using the FlowJo software.

### 2.3 Antigen Presentation Assays

#### 2.3.1 Expression of MHC II Molecules

The *Aire* ^*WT*^ and *Aire* ^*−/−*^ mTECs were cultured as monolayers in DMEM (Gibco, Darmstadt, Germany), supplemented with 10% inactivated foetal bovine serum (FBS) in 75 cm^2^ polystyrene bottles (Corning, New York, NY) in an incubator at 37°C with 5% CO_2_ atmosphere. After acquiring confluence, mTECs were trypsinised and seeded in 12‐well plates and stimulated or not with IFN‐γ (Thermo Fisher Scientific) or LPS (Sigma‐Aldrich, Burlington, MA). Depending on the experiment, the cells were maintained in culture for 3, 4 or 5 days, with different concentrations of IFN‐γ or LPS (0.2, 1.0 and 5.0 ng/μL) and analysed by flow cytometry in an LSRFortessa Cell Analyser (Becton Dickinson, Franklin Lakes, NJ) using the anti‐MHC II antibody.

#### 2.3.2 mTEC Antigen Presentation Assay

*Aire* ^*WT*^ and *Aire* ^*−/−*^ mTECs were cultured in the presence or not of IFN‐γ (1 ng/μL) for 3 days to enhance the surface expression of MHC Class II molecules. Then, cells were γ‐irradiated at 12Gy to stop their proliferation and pulsed, or not, with OVA^323–339^ peptide 10 mg/mL (Sigma‐Aldrich) and cocultured with CD4^+^ T cells purified from splenocytes of OT‐II TCR transgenic mice (B6.Cg‐Tg(TcraTcrb)425Cbn/J mouse, strain # 004194) (The Jackson Laboratories, Bar Harbour, ME). These mice express the mouse alpha‐chain and beta‐chain T cell receptor that pairs with the CD4 coreceptor and is specific for an epitope derived from the chicken ovalbumin (OVA^323–339^ peptide) in the context of I‐A b. The spleen of these mice was aseptically collected and the red blood cells were lysed with ammonium–chloride–potassium (ACK). The cells were enriched with mouse CD4 (L3T4) microbeads (Miltenyi Biotec, Bergisch Gladbach, Germany) for positive selection of mouse CD4^+^ T cells (CD4^+^ T cells purified from OT‐II mice). After bead enrichment, CD4^+^ T cells purified from OT‐II mice were (i) stained with anti‐mouse CD4‐e‐660, anti‐mouse CD8‐FITC and anti‐mouse TCR‐β‐PE (BD Biosciences), (ii) sorted by flow cytometry in a FACS Aria III apparatus (Becton Dickinson, Franklin Lakes, NJ) to obtain a pure population of TCR‐β^+^ CD4^+^ CD8^−^ cells and (iii) stained with carboxyfluorescein succinimidyl ester (CFSE‐CellTrace, Thermo Fisher Scientific). Cells were cocultured in a 96‐well U‐bottom plate with RPMI medium (Gibco), supplemented with 10% inactivated FBS, 1% penicillin and streptomycin, 1% l‐glutamine, 1% sodium pyruvate, 1% HEPES, 25 μL 0.1 M β‐mercaptoethanol. After 3 days, T cell proliferation was evaluated by flow cytometry in an LSRFortessa Cell Analyser (BD) measuring CFSE dye dilution.

#### 2.3.3 mTEC/Dendritic Cell Antigen Presentation Assay

Bead‐enriched CD4^+^ T cells purified from OT‐II mice (2 × 10^3^, 5 × 10^3^ and 10 × 10^3^ cells), containing approximately 35%–40% of TCR‐β^−^ MHC II^+^ CD11c^+^ (BD Biosciences, San Jose, CA) DCs, were cocultured with mTECs, previously pulsed or not, with OVA^323–339^ peptide 10 mg/mL (Sigma‐Aldrich), observing a proportion of 1:10 mTEC: splenocytes. The CD4^+^ T cells purified from OT‐II mice were stained with CFSE, mixed with mTECs pulsed or not with the OVA^323–339^ peptide, co‐stimulated with the anti‐mouse CD28 antibody (BD Biosciences) and cocultured using the procedure as described for the antigen presentation assay.

#### 2.3.4 T Cell Activation Assay Using mTECs and Bone Marrow DCs

For early activation, *Aire* ^*WT*^ and *Aire* ^*−/−*^ mTECs were again cultured with IFN‐γ (1 ng/μL) for 3 days. Cells were then pulsed with the OVA^323–339^ peptide or left unpulsed before coculture with CD4^+^ T cells purified from OT‐II mice.

##### 2.3.4.1 DCs Differentiation

Bone marrow from WT animals was harvested from femurs and tibias and lysed with ACK buffer. Marrow cells were plated in 100 mm polystyrene plates (Corning, Cat #430167) at a density of 2 × 10^6^ cells per plate in RPMI medium supplemented with 10% FBS, 1% antibiotics and antifungal agents and 20 ng/mL GM‐CSF (Thermo Fischer, Peprotech, Cat #315‐03), in a final volume of 10 mL. Cultures were maintained for 7 days, with an additional 10 mL of fresh medium containing 10% FBS, 1% antibiotics and antifungal agents and 40 ng/mL GM‐CSF added on Day 3.

##### 2.3.4.2 OT‐II Lymphocyte Stimulation Assay

Total lymphocytes were isolated from the spleens of naïve OT‐II animals using the MACS CD4 isolation kit (Miltenyi Biotec, Cat #130‐104‐454). After differentiation, DCs were enriched using MACS CD11c microbeads (Miltenyi Biotec, Cat #130–125‐835). mTECs were harvested after 3 days of culture and plated in RPMI medium with 10% FBS and 1% antibiotics and antifungal agents, in co‐culture with OT‐II lymphocytes, mTECs and DCs at a ratio of 2:1:1 (OT‐II:mTEC:DC) in 96‐well U‐bottom polystyrene plates (Corning, Cat #3799). For conditions without DCs, αCD28 (BD Pharmingen, Cat #553295) was added at 1 μg/mL. OVA^323–339^ peptide (Sigma‐Aldrich, Cat #O1641) was used at 10 μg/mL. Negative controls consisted of lymphocytes alone, while positive controls included lymphocytes stimulated with αCD3 (BD Pharmingen, Cat #553057, 2 μg/mL) plus αCD28.

After 18 h of co‐culture, cells were stained with anti‐CD3 FITC (clone 145‐2C11, Invitrogen, Cat #11‐0031‐85), anti‐CD4 APC (clone RM4‐5, BD Pharmingen, Cat #553051) and anti‐CD69 PE‐Cy7 (clone H1.2F3, Biolegend and Cat #104512) monoclonal antibodies. Viability was assessed with the Fixable Viability Stain (FVS; BD Horizon, Cat #65‐0865‐14), incubated at room temperature for 15 min. Cells were then washed with labelling buffer (PBS + EDTA + 0.5% BSA; BD Biosciences, Cat #130‐091‐222 and #130‐091‐376) and incubated with extracellular antibodies for 30 min at 4°C, protected from light. Samples were fixed with 1% paraformaldehyde and analysed on a BD FACSCanto II cytometer. Data analysis was performed using FlowJo v10.8 software (Becton Dickinson and Company, Franklin Lakes, NJ).

### 2.4 Transcriptome Analysis

The mTEC raw RNA‐sequencing data were obtained from our previous study [32], accessible on the Gene Expression Omnibus (https://www.ncbi.nlm.nih.gov/geo/) (PRJNA763914). The raw FASTQ sequences were analysed through the FASTQC programme (https://www.bioinformatics.babraham.ac.uk/projects/fastqc/). The FASTQ sequences were mapped to the *M. musculus* reference genome (mm21) using the STAR 2.5 Spliced Aligner programme (https://github.com/alexdobin/STAR). The latter programme outputs a BAM file containing the sequences and their genomic references and a GTF file with gene annotations used to determine the number of reads per transcript through the HTSeq Count programme (http://htseq.readthedocs.io).

We obtained a list of transcripts for each RNA sample that served as input for determinations of the differentially expressed (DE) mRNAs through the Rleave [37] algorithm within the R platform (https://www.r‐project.org). Rleave is an in silico cross‐validation protocol for DE transcript analysis, permitting normalisation of the transcript variability by combining a conventional and a Leave‐one‐out analysis. We filtered the mRNAs based on their fold change (FC). We defined *Aire* ^*WT*^ as the reference and DE mRNAs were those with *p*‐values < 0.05, false discovery rates (FDRs) corrected using the Benjamini–Hochberg method and FC ≥ 1.5. The DE mRNAs were used to construct heat maps.

### 2.5 Differentially Expressed mRNAs

The DE mRNAs were analysed in terms of functional enrichment through the Database for Annotation, Visualisation and Integrated Discovery (DAVID) annotation tool (https://david.ncifcrf.gov/) for the identification of the pathways related to antigen processing and antigen presentation. A functional category was considered significant if it comprised at least five mRNAs and scored *p* < 0.005 with Benjamini–Hochberg correction. The algorithm Search Tool for the Retrieval of Interacting Proteins (STRING) database (https://string‐db.org/) was used to construct networks, establish interactions among the genes and encode validated protein data available in the literature.

### 2.6 RNA Isolation and Real‐Time PCR Analysis

Total RNA was extracted from *Aire* ^*WT*^ or *Aire* ^*−/−*^ mTECs treated, or not, with IFN‐ γ, using RNeasy (QIAGEN, Hilden, Germany), according to the manufacturer's instructions. All RNA samples were recovered in 14 μL of nuclease‐free H_2_O and quantified using the Nanodrop ND‐1000 apparatus (Thermo Fisher Scientific) and analysed regarding their integrity through microfluidic electrophoresis in a Bioanalyser 2100 apparatus (Agilent).

Synthesis of cDNA was performed from 10 ng/μL of total RNA prepared as described above in a 20 μL final volume using SuperScript III Reverse Transcriptase cDNA Synthesis kit for RT‐qPCR (Thermo Fisher Scientific), according to the manufacturer's instructions. The PCR programme run (65°C, 5 min; 4°C, 1 min; 25°C, 5 min; 50°C, 30 min; 70°C, 15 min) was performed in a Bio‐Rad T100 Thermal Cycler (Bio‐Rad, Hercules, CA). Real‐time PCR was then used to semi‐quantify *Ifnr1, Ifnr2, Ciita.pI, Ciita.pIII, Ciita.pIV, Irgb6, Irgm1* and *Irgm3* mRNA expression levels with the iTaq Universal SYBR Green Supermix (BioRad), following the manufacturer's instructions. As a reference for constitutive gene expression, we used Gapdh primers (Thermo Fisher Scientific).

For the quantification of mRNA expression levels, the reaction was performed in a final volume of 10 μL containing 0.2 μM of each specific primer: (i) *Gapdh* forward: AAG GGC TCA TGA CCA CAG TC, *Gapdh* reverse: CAC ATT GGG GGT AGG AAC AC; (ii) *Ciita‐pI* forward: ACA GGG ACC ATG GAG ACC ATAG, *Ciita‐pI* reverse: GGG TCG GCA TCA CTG TTA AGG; (iii) *Ciita‐pIII* forward: GCC GGA GTT GCA AGA CCA TAG, *Ciita‐pIII* reverse: GGG TCG GCA TCA CTG TTA AGG; (iv) *Ciita‐pIV* forward: GAG ACT GCA TGC AGG CAG CAC, *Ciita‐pIV* reverse: GGG TCG GCA TCA CTG TTA AGG; (v) *Irgm3* forward: CTG AGC CTG GAT TGC AGC TT, *Irgm3* reverse: GTC TAT GTC TGT GGG CCT GA; (vi) *Irgb6* forward: TTG CCA CCA GAT CAA GG TCA C, *Irgb6* reverse: CAA GGT GAT GTC ATA TTC AGA GAT G; (vii) *Irgm1* forward: CTC TGG ATC AGG GTT TGA GGA GTA, *Irgm1* reverse: GGA ACT GTG TGA TGG TTT CAT GAT A; (viii) *Ifnγr1* forward: CTT GAA CCC TGT CGT ATG CTG G, *Ifnγr1* reverse: TTG GTG CAG GAA TCA GTC CAG G and (ix) *Ifnγr2* forward: CCT TCC AGC AAT GAC CCA AGA C, *Ifnγr2* reverse: TGT GAT GTC CGT ACA GTT CGG C, iTaq Universal SYBR Green Supermix (BioRad) and 100 ng/μL of the newly‐synthesised cDNA. The PCR programme conditions included: (i) denaturation at 95°C, 3 min, (ii) 39 cycles for amplification (95°C, 10 s; 60°C, 30 s) and (iii) a final melt curve increment of 0.5°C from 65°C to 95°C for 5 s. The reaction was performed in a CFX96 Touch Real‐Time PCR Detection System (BioRad). We analysed real‐time PCR data using the comparative threshold cycle (CT) method. Individual relative gene expression values were calculated using the following formula: Δ*C*_T_ = *C*_T_(target gene) − *C*_T_(reference gene). Experiments were performed on at least three independent replicates.

### 2.7 Lysosomal Functional Analyses

The expression of cathepsin L (CTSL) and the total lysosome [38] was measured using the Magic Red CTSL (ImmunoChemistry Technologies, San Jose, CA) and Lysosome‐Specific (Biovision, Cambridge, UK) kits, respectively, following the manufacturer's instructions. Briefly, cells were stimulated or not with IFN‐γ(1 ng/μL) for 3 days and then incubated with the solution containing the fluorogenic substrate for CTSL or with the specific self‐quenched substrate for lysosomes for 1 h at 37°C in DMEM (10% FBS). The cells were then washed and analysed by flow cytometry. In cells with active CTSL, the substrate exhibits red fluorescence after proteolytic cleavage and in those with lysosomal activity, the fluorescence is green. The fluorescent signal is proportional to intracellular lysosomal activity in cells with active lysosomes.

### 2.8 Statistical Analyses

Depending on the distribution of the variables in each comparison, statistical analyses were performed using the unpaired *t* test, considering significant *p*‐values < 0.05 and performed using GraphPad software (Version 6.0, GraphPad Software Inc., La Jolla, CA). All experiments were performed in at least three replicates.

## 3 Results

### 3.1 The Absence of *Aire* Modulates Genes Involved With Antigen Processing and Presentation

Revisiting the mTEC RNA sequencing data from our previous work [32], we meticulously evaluated the differential expression of MHC I and II antigen processing and antigen presentation gene pathways. The comparisons of the transcriptional profile between *Aire* ^*WT*^ and *Aire* ^*−/−*^ mTECs under spheroid format revealed 23 modulated genes involved with antigen processing and antigen presentation pathways, including the upregulated (*Rab34, Il33, Rab32, Bcar1, Atg5, Calr*) and the down‐regulated (*Cd74, H2‐T22, H2‐K1, H2‐M3, H2‐Q4, Tap2, Tap1, Ifi30, Psmb8, Tapbp, Bag6, Mfsd6, H2‐Dmb1, Psap, B2m, Tmem106a, Havcr2*) in *Aire* ^−/−^ comparing to *Aire* ^*WT*^ mTECs (Figure S1A).

The construction of a protein interaction map revealed that Aire, Ifn‐γ and Ciita interact with many proteins involved in antigen processing and presentation. Aire primarily interacts with IFN‐γ, which further interacts with CD74 and Ciita; however, the CD74 protein makes the major node in the protein network, encompassing antigen presentation and processing molecules, which are DE between *Aire* ^*WT*^ and *Aire* ^*−/−*^ cells (Figure S1B).

### 3.2 *Aire* Controls IFN‐ γ‐Dependent MHC II Expression in mTECs

**FIGURE 1:**
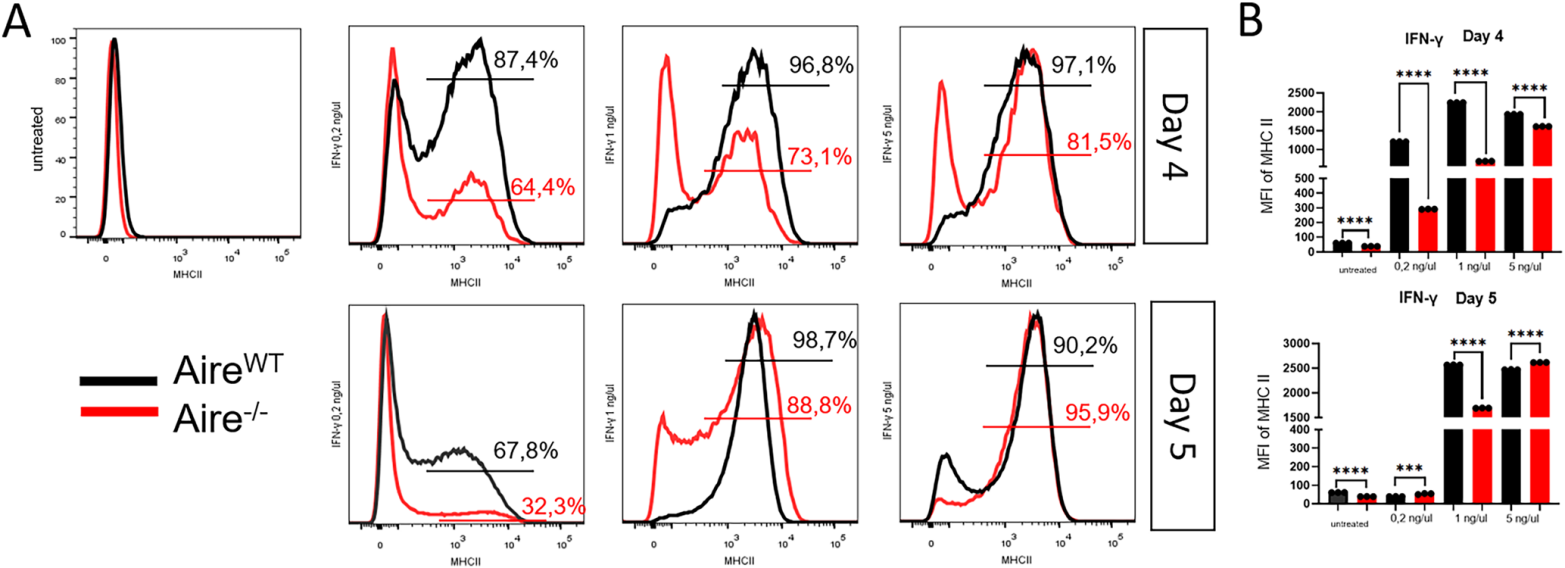
Expression profile of MHCII in mTECs. (A) Flow cytometry analysis of expression of MHC II in mTECs *Aire* ^*WT*^ and Aire^−/−^untreated or after 4 or 5 days of treatment with IFN‐γ (representative figure from three independent determinations). The percentage of MHC class II positive cells are shown in the figure. (B) Median fluorescence intensity in MHCII positive fraction was calculated by Prism GraphPad and represented in the graphic bar beside. Data shown (mean ± SD) are from three independent determinations and the significant difference between *Aire* ^*WT*^ and *Aire* ^*−/−*^ was analysed by the unpaired *t* test. The *Aire* ^*WT*^ cells are represented in black and the *Aire* ^*−/−*^ in red.

In the steady state, *Aire* ^*WT*^ or *Aire* ^−/−^ mTECs did not express MHC II molecules (Figure 1A). To induce the MHC II expression, we stimulated both cell types with IFN‐γ or LPS [24] in different concentrations for 4 and 5 days and then measured the MHC II surface expression. After stimulation with IFN‐γ at 0.2 ng/uL for 4 days, we observed an increased MHC II expression in both *Aire* ^*WT*^ and *Aire* ^−/−^ mTECs (Figure 1B). Nonetheless, this difference is not detected by extending the culture for 5 days, probably related to the lower amount of IFN‐γ and an extra day of culture.

The *Aire* ^*−/−*^ showed a significantly impaired induction of MHC II expression compared to *Aire* ^*WT*^ and this difference persisted even with higher IFN‐γ doses (1 or 5 ng/μL). Nonetheless, when both cell lines were stimulated with LPS, no differences in MHC II expression were observed at days four or five, regardless of the dose (5 or 50 ng/mL). This result suggests that LPS did not induce MHC II expression in either cell line (Figure S1C,D). Therefore, both *Aire* ^*WT*^ and *Aire* ^−/−^ mTECs increased MHC II expression in an IFN‐γ dose‐dependent manner; however, the *Aire* ^*−/−*^ cell line exhibited a delayed MHC II expression.

### 3.3 *Aire^WT^* or *Aire* ^*−/−*^ mTECs Need DCs to Complete Antigen Presentation

**FIGURE 2:**
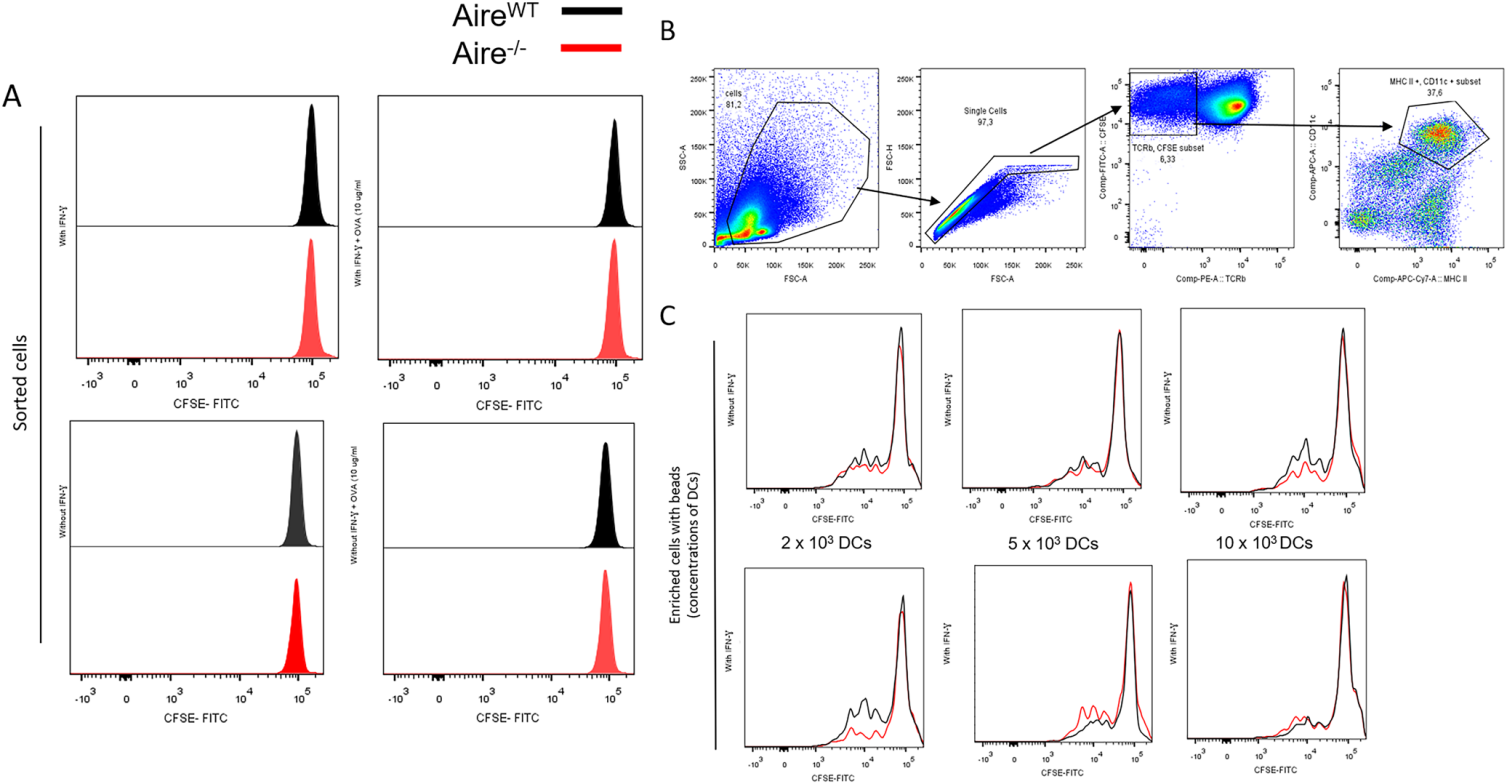
FACS analysis of T‐cell proliferation (T‐cell assay). Proliferative responses of CFSE‐labelled, ovalbumin (OVA)‐specific, TCR‐transgenic CD4^+^ OT‐II T cells cultured in vitro with *Aire* ^*WT*^ or *Aire* ^*−/−*^ mTECs and/or DCs pulsed with 10 μg/mL OVA^323–339^ peptide, with or without IFN‐γ treatment. The figure depicts conditions using varying numbers of DCs (2 × 10^3^, 5 × 10^3^ or 10 × 10^3^) co‐cultured with T cells. Data were collected after 3 days of culture. (A) Representative graphic of the experiment with sorted CD4^+^ T cells from the spleen with *Aire* ^*WT*^ or *Aire* ^*−/−*^ cells. (B) Gating strategy to confirm the phenotypic profile of DCs population. The lines indicate the sense: SSC‐A × FSC‐A → FSC‐H × FSC‐A → CFSE × TCRB → CD11c × MHCII. (C) Representative graphic of the experiment with bead enrichment of CD4^+^ T cells from spleen with *Aire* ^*WT*^ or *Aire* ^*−/−*^ cells pulsed with OVA^323–339^ peptide, with different concentrations of APC: in the first 2 × 10^3^, in the second 5 × 10^3^ and in the third 10 × 10^3^. The co‐culture with mTECs and DCs not pulsed with OVA^323–339^ peptide was performed, showing no T cell proliferation (data not shown in the manuscript). The *Aire* ^*WT*^ cells are represented in black and the *Aire* ^*−/−*^ in red. The figures are representative of at least three experiments.

To evaluate the potential antigen presentation of the mTEC cells studied, CD4^+^ T cells purified from OT‐II mice splenocytes (TCR‐β^+^ CD4^+^ CD8^−^), bearing a specific TCR for the OVA^323–339^ peptide, were separated by flow cytometry and co‐cultured with *Aire* ^*WT*^ or *Aire* ^*−/−*^ mTECs, pre‐treated or not with IFN‐γ. Using these purified co‐cultures, we did not observe the proliferation of CD4^+^ T cells purified from OT‐II mice, indicating that mTECs, without help, could not induce T cell proliferation (Figure 2A). To further evaluate the mTEC antigen presenting potential in cooperation with DCs, we repeated this assay by coculturing *Aire* ^*WT*^ or *Aire* ^*−/−*^ mTECs with splenic OTII TCR transgenic CD4^+^ T cells in the presence of DCs (TCR‐β^−^ MHCII^+^ CD11c^+^) (Figure 2B), at different concentrations (Figure 2C). Unlike what was previously observed with mTECs alone, in these conditions, T cells proliferated once cocultured with mTECs and DCs, independently of their concentration (Figure 2C and S2A). However, we did not observe differences when comparing cells treated or not with IFN‐γ or between *Aire* ^*WT*^ and *Aire* ^*−/−*^ in these conditions (Figure S2B). As such, these results demonstrated that *Aire* ^*WT*^ or *Aire* ^*−/−*^ mTECs cannot support T cell proliferation, even when expression of MHC II is induced in these cells. Furthermore, neither *Aire* ^*WT*^ nor *Aire* ^*−/−*^ mTECs significantly affected the capacity of DCs to induce T cell proliferation in response to a specific antigen.

### 3.4 *Aire^WT^* and *Aire* ^*−/−*^ mTEC Induce T Cell Activation

**FIGURE 3:**
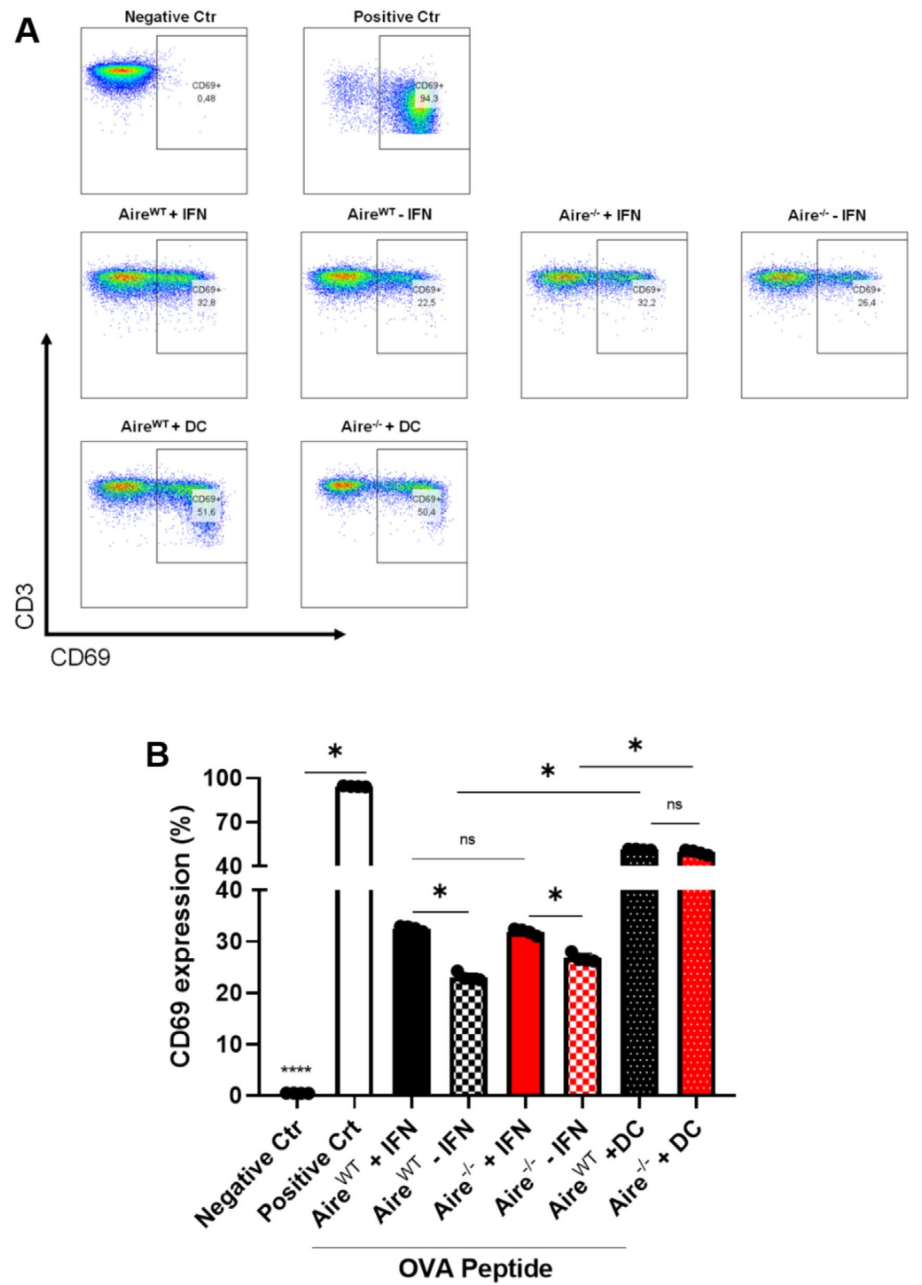
T cell activation assay‐flow cytometry analysis for the CD69 marker after 18 h of culture. (A) In first line, as negative control co‐culture with DCs and splenocytes without OVA^323–339^ peptide. As positive control, co‐culture with DCs and splenocytes in the presence of the OVA peptide (10 μg/mL). In the second line, co‐culture with mTEC *Aire* ^*WT*^ or mTEC *Aire* ^*−/−*^ and splenocytes with OVA^323–339^ peptide (10 μg/mL), stimulated or not with IFN‐γ (1 ng/μL). In the third line, co‐culture with mTEC *Aire* ^*WT*^ or mTEC *Aire* ^*−/−*^ and splenocytes with OVA^323–339^ peptide (10 μg/mL) stimulated with IFN‐γ (1 ng/μL) in the presence of DCs. The figures are representative of at least four experiments. (B) Median fluorescence intensity in CD69‐positive fraction was calculated by Prism GraphPad and represented in the graphic bar beside. Data shown (mean ± SD) are from four independent determinations and the significant difference between *Aire* ^*WT*^ and *Aire* ^*−/−*^ was analysed by the unpaired *t* test. The *Aire* ^*WT*^ cells are represented in black and the *Aire* ^−/−^ in red.

T cells cocultured with bone marrow DCs (DC/T) and stimulated with OVA^323–339^ peptide exhibited robust CD69 expression (94.3%) after 18 h in culture (Figure 3A, top panel). In contrast, cocultures of OT‐II splenocytes with *Aire* ^*WT*^ (22.5%) or *Aire* ^*−/−*^ (26.4%) mTECs showed only modest CD69 induction in T cells in the absence of IFN‐γ stimulation (Figure 3A, middle panel). Upon addition of OVA^323–339^ peptide together with IFN‐γ, CD69 expression increased in T cells when cocultured with *Aire* ^*WT*^ (32.8%) and *Aire* ^*−/−*^ (32.2%) (Figure 3A, middle panel). On the other hand, both *Aire* ^*WT*^ (51.6%) and *Aire* ^*−/−*^ (50.4%) treated with IFN‐γ and cocultured with DC/T cell induced a higher CD69 expression in T cells, indicating that mTECs, regardless of *Aire* status, promoted limited T‐cell activation through a mechanism independent of MHC II presentation. Notably, *Aire* ^*WT*^ and *Aire* ^*−/−*^ mTEC lines cultured without IFN‐γ did not express MHC II (Figure 1), yet still induced CD69 expression in cocultured T cells. As a control, cultures without OVA^323–339^ peptide showed no CD69 induction (Figure S3B,C). Additional negative (OT‐II only) and positive (OT‐II with αCD3 and αCD28) controls were included. The gating strategy is presented in Figure S3A.

### 3.5 *Aire* Controls the IFN‐γ‐Mediated Expression of Genes Associated With the MHC II Pathway

**FIGURE 4:**
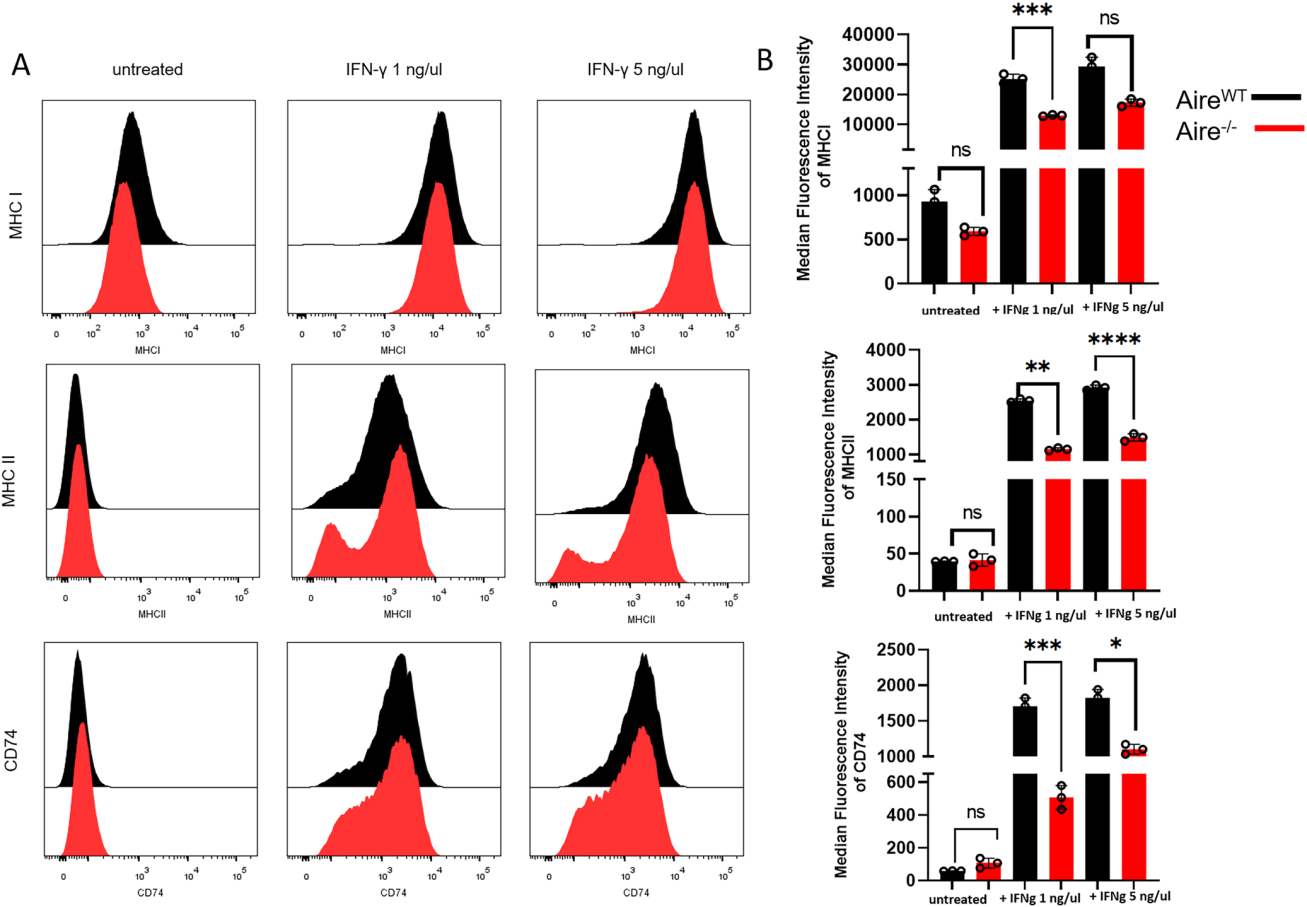
Expression profile of MHCI, MHCII and CD74 in mTECs. (A) Flow cytometry analysis of expression of MHC I, MHCII and CD74 in mTECs *Aire* ^*WT*^ and *Aire* ^*−/−*^ untreated or after 3 days of treatment with IFN‐γ in different concentration (1 and 5 ng/μL) (representative figure from at least three independent determinations). (B) Median fluorescence intensity was calculated by Prism GraphPad and represented in the graphic bar beside. Data shown (mean ± SD) are from three independent determinations and the significant difference between *Aire* ^*WT*^ and *Aire* ^*−/−*^ was analysed by the unpaired *t* test. The *Aire* ^*WT*^ cells are represented in black and the *Aire* ^*−/−*^ in red.

**FIGURE 5:**
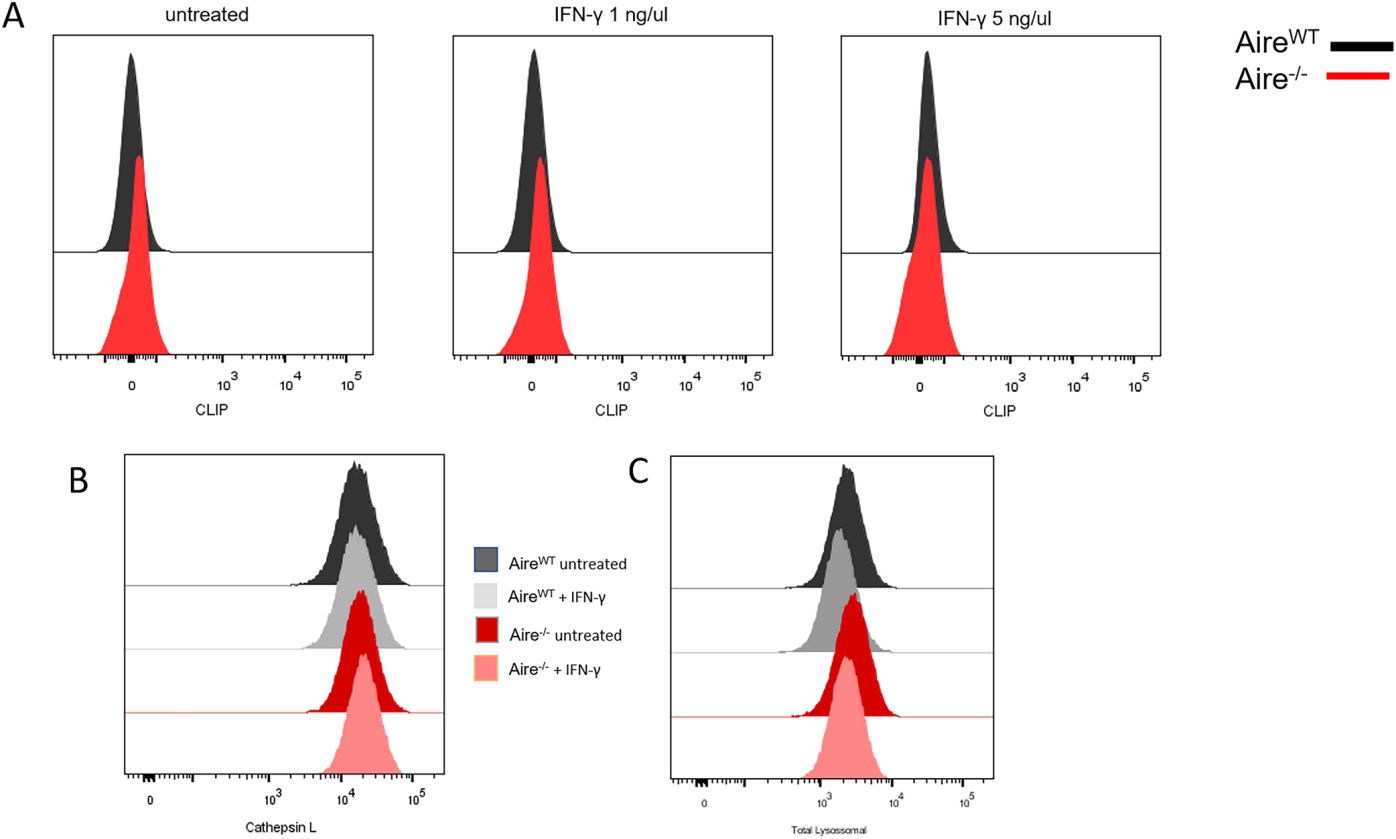
Flow cytometry analysis of molecules associated with antigen processing. (A) 15G4 (intermediate clip) in *Aire* ^*WT*^ and *Aire* ^*−/−*^ mTECs untreated or after 3 days of treatment with 1 and 5 ng/μL IFN‐γ (representative figure of three independent determinations). (B) Histograms showing representative assessments of cathepsin L (CTSL, responsible for cleaving the invariant chain) activity in untreated *Aire* ^*WT*^ (black) and untreated *Aire* ^*−/−*^ (red), *Aire* ^*WT*^ + INFγ (grey) and *Aire* ^*−/−*^ + INFγ (rose). (C) Histograms showing representative assessments of total lysosomal activity in untreated *Aire* ^*WT*^ (black) and untreated *Aire* ^*−/−*^ (red), *Aire* ^*WT*^ + INFγ (grey) and *Aire* ^*−/−*^ + INFγ (orange). The figures are representative of at least three experiments.

Considering that *Aire* influenced the IFN‐γ‐driven expression of MHC II, we further evaluated whether critical elements involved with the MCH II pathway were affected by *Aire* deficiency. We co‐analyse the surface expression of MHC II, the intracellular CD74 (invariant chain of the MHC molecule) and MHC I expression. The MHC I expression increased after IFN‐γ stimulation, but its expression was decreased in *Aire* ^*−/−*^ mTECs in the steady state as well as after IFN‐γ stimulation. Similarly, when cells were treated with IFN‐γ (1 or 5 ng/μL), the expression of MHC II and CD74 was increased in both mTECs (Figure 4A,B); however, part of the *Aire* ^*−/−*^ mTEC showed a limited induction of MHC II and CD74 molecules upon IFN‐γ treatment (Figure 4A). Notably, the expression of the intermediate clip of the invariant chain (CLIP), which is expressed in vivo in cTECs [38], was not observed for both cell lines (*Aire* ^*WT*^ and *Aire* ^*−/−*^) (Figure 5A). During antigen processing, cathepsins and lysosome proteins degrade antigens to be subsequentially presented by MHC II molecules. Then, we analysed whether the absence of Aire could interfere with the concentration of these proteins. Figure 5B,C show that both cell lines have closely similar protein concentrations of cathepsin L (expressed in cTECs and mTECs) and total lysosomal proteins. Therefore, the absence of *Aire* apparently does not interfere with the lysosomal stage of antigen processing. Our results corroborated RNA sequencing analysis and showed that *Aire* deficiency broadly impacts genes involved in the MHC II antigen presentation/processing pathway. However, it does not interfere with the functionality of mTECs.

### 3.6 *Aire* Deficiency Impacts on IFN‐γ Driven Expression of MHC II Transactivators

**FIGURE 6:**
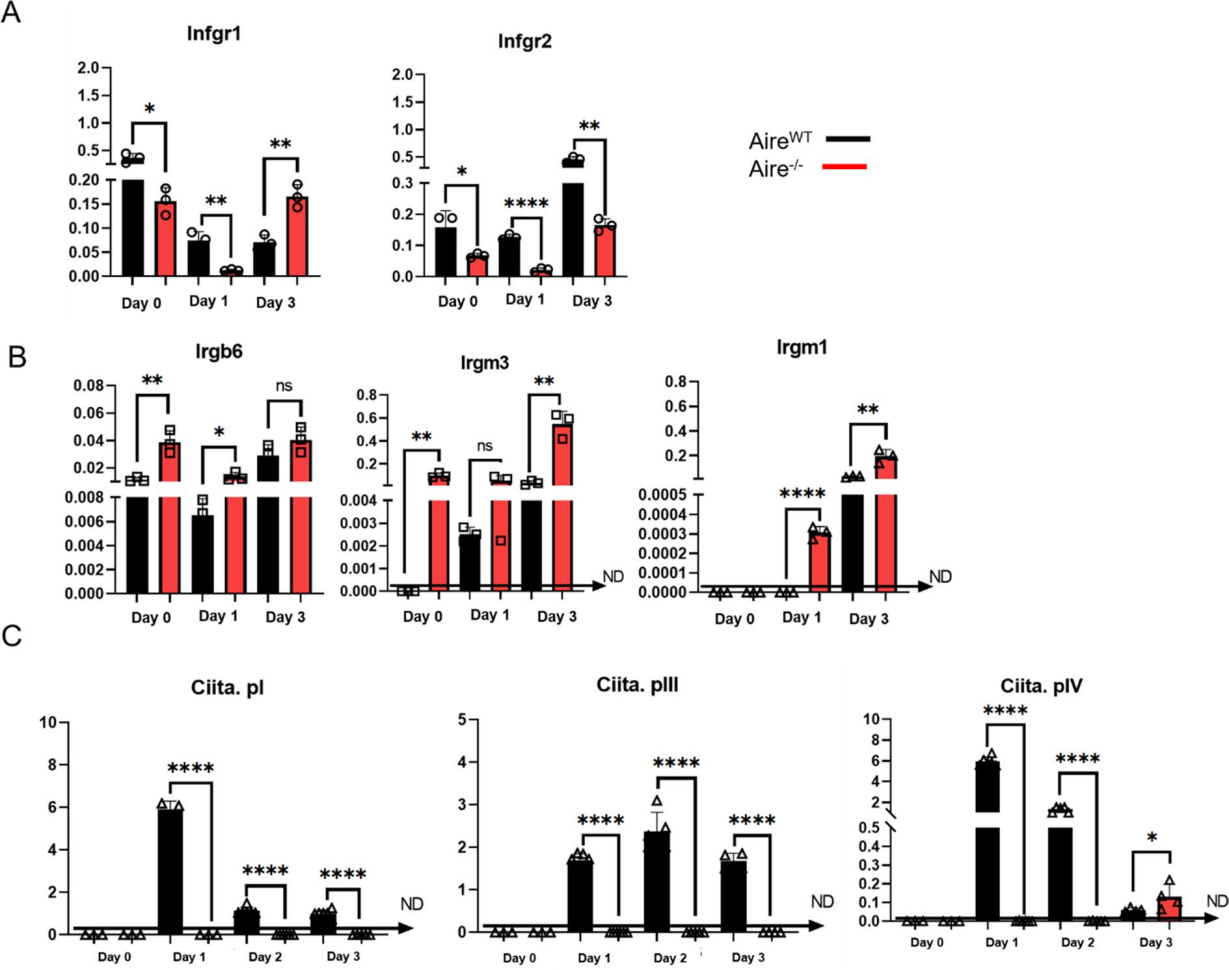
Relative fold change determined by quantitative real‐time PCR (qRT‐PCR) analysis of antigen presentation genes and INFγ signalling genes. Evaluation of the relative expression level of the mRNAs: (A) *IFNgR1, IFNgR2*; (B) *Irgb6, Irgm1, Irgm3*; (C) *Ciita.pI, Ciita.pIII, Ciita.pIV* in *Aire* ^*WT*^ and *Aire* ^*−/−*^ cells during 0, 1 and 3 days of treatment with IFN‐γ. All data were normalised with *GAPDH* expression and given as relative to control. Data were represented as mean ± SD and difference between groups was analysed by unpaired *t* test (*p* ≤ 0.05). The *Aire* ^*WT*^ cells are represented in grey and the *Aire* ^*−/−*^ in red. The figures are representative of at least three experiments. ND = not detected.

To confirm that the delayed MHC II expression in *Aire* ^−/−^ cells was not due to an impaired response to IFN‐γ stimulation, we evaluated the gene expression of IFN‐γ receptors and GTPases that IFN‐γ induces. As shown in Figure 6A, both cell lines expressed the IFN‐γ receptors 1 and 2 (*Ifnγr1* and *Ifnγr2*). *Aire* ^*WT*^ cells showed a reduction in *Ifnγr1* expression at Days 1 and 3 compared with Day 0, whereas *Aire* ^*−/−*^ cells maintained elevated receptor levels after 3 days of IFN‐γ stimulation (with only a slight decrease at Day 1). In addition, both cell lines upregulated *Ifnγr2* in response to IFN‐γ, with a more pronounced increase observed in *Aire* ^*WT*^ cells. The GTPases *Irgb6, Irgm1* and *Irgm3* are expressed in both cell lines. Although GTPases were increased in *Aire* ^*−/−*^, both cells similarly responded to IFN‐γ stimulation (Figure 6B).

In mice, the *Ciita* gene is controlled by three promoters (pI, pIII and pIV), expressing the *Ciita* types I, III and IV [39, 40]. We observed that *Aire* ^*WT*^ cells express the *Ciita* promoters after the stimulation of IFN‐γ for 1 and 3 days; however, the *Aire* ^−/−^ does not express *Ciita* after IFN‐γ stimulation, except for isoform IV at Day 3 (Figure 6C). These results indicate that the loss of *Aire* could interfere with the expression of genes necessary for antigen processing and presentation pathways, potentially impairing the negative selection process.

## 4 Discussion

MHC II‐driven antigen presentation by mTECs is crucial for T cell maturation and central tolerance within the thymus. It ensures that developing T cells recognise and appropriately respond to self‐antigens, while eliminating cells that could lead to aggressive autoimmunity [1, 2]. In APS‐1, the increased IFN‐γ production and its downstream signalling effects affect self‐tolerance mechanisms that AIRE [41, 42] can mediate. Amid this intricate landscape, IFN‐γ emerges as a potent modulator of MHC II expression and self‐antigen presentation.

Because we have previously reported that *Aire* ^*−/−*^ cells exhibited several abnormalities regarding the mTEC‐thymocyte [31] and mTEC‐mTEC adhesion [32] and considering that cell adhesion is an essential process for mTEC antigen presentation, in this study, we hypothesised that *Aire* ^*−/−*^ cells may inefficiently present antigens. Taking advantage of transcriptomic data from *Aire* ^*WT*^ and *Aire* ^−/−^ spheroids from our previous study [32], we observed several genes modulated by *Aire*, including classical class I (*H2‐Q4*), non‐classical class I (*H2‐T22*), extended class I (*H2‐M3*), extended class II (*H2‐K1*) genes, together with genes related to antigen processing (*Tap1, Tap2, H2‐Dmb1* and *the invariant chain CD74*).

In this study, we observed that *Aire* ^*WT*^ and *Aire* ^−/−^ cells did not constitutively express MHC II molecules. Considering that IFN‐γ is a potent regulator of MHC II expression and because its effects are crucial for antigen presentation and for the modulation of immune responses [43, 44], we stimulated both cell lines with IFN‐γ or LPS, observing that only IFN‐γ induced the expression of MHC II molecules. Nonetheless, the mutant *Aire* ^−/−^ exhibited a delayed expression of MHC II even using different concentrations of IFN‐γ, supporting the hypothesis that *Aire* may influence MHC II expression. Although the mTEC cell line used in this study presents an immature phenotype as shown in our previous study [32], the delayed response to IFN‐γ observed in the *Aire* ^*−/*−^ cell line highlights a possible regulatory role of *Aire* in modulating MHC class II expression via *Ciita*. Although this delay may not fully recapitulate the in vivo scenario, where MHC II is constitutively expressed in mTECs even in the absence of *Aire*, it provides a model to further investigate the mechanisms underlying *Aire*‐dependent regulation of antigen presentation in the thymic epithelium.

Noteworthy, autoimmune manifestation in APS‐1 patients does not manifest simultaneously after delivery, as occurs with the complete MHC II deficiency [42], but appears along life [13]. In this context, a delayed expression of MHC II molecules may produce cumulative tolerance losses, allowing the appearance of autoimmune disorders throughout life. Next, we proceeded with the antigen presentation assay, using a transgenic mouse (OT‐II) exhibiting a specific TCR that recognises the OVA^323–339^ peptide. To evaluate the direct antigen presentation, we sorted CD4^+^ T cells purified from OT‐II mice splenocytes (without DCs, as checked by flow cytometry), observing that *Aire* ^*WT*^ and *Aire* ^−/−^ cells did not present the peptide (Figure 2A). Former studies did not mention whether or not mTECs were contaminated with other antigen‐presenting cells [24, 25, 26]; however, more recent studies evaluating mouse‐isolated mTECs have shown an indisputable role of the direct antigen presentation [45, 46]. Nonetheless, in the absence of direct presentation, the concept of cooperative antigen transfer [17] exists, in which mTECs transfer antigens to the DCs. It is well established that DCs are potent professional antigen‐presenting cells and therefore strong inducers of T‐cell proliferation. Thus, the presence of ‘only DCs’ naturally triggers a robust T‐cell proliferative response. In contrast, mTECs play a more modulatory role in thymic selection and immune tolerance and their effects on T‐cell proliferation are more subtle and dependent on environmental thymic mediators, which, eventually, are not present in the experimental assay. The observation of relatively lower proliferation in the coculture condition does not refute the importance of mTECs, but rather reflects their physiological role in shaping T‐cell responses and maintaining tolerance, potentially by limiting excessive proliferation or promoting the generation of regulatory phenotypes.

To evaluate the mTEC presentation with the participation of DCs, we initially used T CD4^+^ enriched from splenocytes containing spleen DCs in different concentrations, observing that both cells proliferated in response to the OVA^323–339^ peptide; however, without differences between *Aire* ^*WT*^ and *Aire* ^−/−^ cells (Figure 2B,C). Several possibilities may explain this finding, including (i) DCs were obtained from an *Aire* sufficient mouse, supplementing the *Aire* deficiency and (ii) the presence of heterogeneous mTEC subsets, expressing high or low levels of MHC II molecules [44, 47], (iii) the mTECs evaluated in this study exhibit a limited ability to present antigen by themselves.

Although mTECs only presented the antigen in the presence of DCs contaminated from splenocytes, we further evaluate the ability of mTECs to activate T cells (Figure 3A–C) in the presence or absence of DCs derived from bone marrow. Indeed, both mTECs (*Aire* ^*WT*^ and *Aire* ^−/−^) induced T cell activation (CD69 marker expression) in a process that is independent of MHC II presentation and of the presence of *Aire*. This finding may be explained by the following reasons: (i) besides the delay on MHC‐II molecule expression (first signal), there may be a decreased or lack of co‐stimulatory signals (second signal) from mTECs to splenocytes; (ii) the regulatory role of mTECs on inducing tolerance rather than T cell activation. In this case, it is worth mentioning that the regulatory cells may also express CD69 [48]; (iii) limitation of our experimental model; that is, this is a somehow artificial model system to explore antigen presentation and/or T cell activation, in this case, mediated by mTECs in cooperation with DCs. The artificiality of the model is relative to the use of transgenic cells clonally expressing a TCR that recognises a foreign specific OVA^323–339^ peptide. Note that in physiological conditions, the antigens in thymus are the self‐peptides expressed by mTECs. However, the TCR/OVA is the state‐of‐the‐art model available nowadays for this type of evaluation, which is much different from the polyclonality of mTECs.

Although no differences were observed for the MHC I molecules related to *Aire* deficiency, we needed to further understand the delay in MHC II expression, which occurred together with the delay in expression of the CD74 invariant chain (Figure 4A,B). The invariant polypeptide of the major histocompatibility complex is a type II integral membrane protein, acting as a chaperone for MHC II protein expression, fixing to the peptide‐binding site of the MHC class II alpha/beta heterodimers and forming an alpha‐beta‐CLIP complex [49]. CD74 also plays a vital role in thymic positive and negative selection, especially in the maturation of mTECs [50, 51]. In addition to these well‐described roles in the thymus, our data show that the absence of *Aire* causes a delay in the CD74 expression after INF‐γ stimulation, indicating that *Aire* is important not only for antigen presentation but also for antigen processing by MHC II molecules.

Besides CD74, other processes are needed for the efficient antigen presentation via MHC II molecules, including the enzymatic cleavage of the invariant chain, the removal of the clip from the MHC II groove and the exchange of the clip by the peptide to be presented to the specific CD4^+^ lymphocyte. Because most of the antigen processing machinery occurs in the endolysosome, we evaluated the total lysosome expression, the specific cathepsin L and the levels of the intermediate clip (Figure 5A–C). The levels of the intermediate clip did not differ in *Aire* ^*WT*^ and *Aire* ^−/−^cells. Similarly, the cathepsin L and the total lysosomal expression did not differ between *Aire* ^*WT*^ and *Aire* ^−/−^ cells. Furthermore, the expression of the *H2‐Dmb1* gene was repressed in the *Aire* ^−/−^ cells when compared to the *Aire* ^*WT*^ mTECs. These results indicate that most of the lysosomal‐dependent antigen processing pathway is not influenced by *Aire*, exception made to the *H2‐Dmb1* gene, which was downregulated in the transcriptome analysis. As a result, the delay in MHC II expression was not dependent on this lysosomal intermediate pathway.

We further studied whether or not *Aire* influences the IFN‐γ‐mediated expression of MHC molecules by evaluating the expression of IFN‐γ receptors, the IFN‐γ‐inducible immunity‐related GTPases (IRGs) and the MHC II transactivators (Figure 6A–C). Although both *Aire* ^*WT*^ and *Aire* ^*−/−*^ mTECs responded to IFN‐γ stimulation, their transcriptional profiles revealed notable differences in IFN‐γ receptors and IFN‐responsive genes. *Aire* ^*−/−*^ cells exhibited a delayed upregulation of *Ifnγr1* and sustained higher expression of several IFN‐inducible GTPases (including *Irgb6, Irgm1 and Irgm3*), suggesting a more prolonged or amplified cytokine response. In contrast, *Aire* ^*WT*^ cells displayed an earlier but transient activation pattern, with progressive downregulation of *Ifnγr1*, consistent with rigid regulatory control [52]. These divergent dynamics may shape downstream events such as antigen processing and presentation, particularly the timing and amplitude of Ciita isoform expression and MHC II upregulation. The absence of Aire may therefore compromise not only the transcription of TRAs but also modulate mTEC sensitivity to inflammatory mediators, ultimately altering their contribution to central tolerance. This divergence underscores *Aire's* broader role in fine‐tuning mTEC function within inflammatory contexts, extending beyond its classical role in TRA regulation.

Finally, we evaluated the expression of the MHC II transactivator (Ciita, isoforms pI, pIII and pIV). We unexpectedly observed the expression of *Ciita isoforms I and III* in *Aire* ^*WT*^ mTECs in response to IFN‐γ stimulation, a finding not previously reported for mTECs. On the other hand, *Ciita isoform IV* was upregulated in *Aire* ^*WT*^ mTECs. Considering that (i) the absence of *Aire* decreased the expression of all *Ciita* isoforms after IFN‐γ stimulus, (ii) Ciita increases the expression of both MHC II [53] and CD74 [54] molecules, the decreased induction of *Ciita* in response to IFN‐γ stimulation may be responsible for the delayed upregulation of MHC class II expression. In summary, this study showed that *Aire* ^*WT*^ and *Aire* ^−/−^ mTECs present antigens only in the presence of CD4^+^ T cells purified from OT‐II mice lymphocytes and DCs. Although *Aire* ^−/−^ mTECs similarly presented antigens, we observed delayed expression levels of MHC II and the invariant chain (CD74) molecules on the cell surface. Both cell lines responded to IFN‐γ stimulus when evaluated using IFN‐γreceptors and IRGs. However, *Aire* ^−/−^ cells exhibited a decreased expression of the MHC II transactivator (Ciita), which modulated MHC II and CD74 molecules. The lysosomal and the cathepsin expression are preserved in both mTECs. Therefore, *Aire* is involved in several steps of antigen processing (*CD74, H2‐dmb1*) and antigen presentation (*MHC II, Ciita*).

In this murine mTEC *Aire* deficiency model, we observed that critical steps of antigen processing and presentation were affected, including the delayed expression of MHC II, *Ciita* isoforms and CD74. Mutant or WT mTECs alone did not present antigens in the experimental setting used, but induced CD69 expression. Antigen presentation to T lymphocytes occurred only in the presence of DCs. In the human counterpart of *AIRE* deficiency (APS‐1), the random appearance of chronic mucocutaneous candidiasis, endocrine and non‐endocrine autoimmune disorders, together with the development of distinct autoantibodies directed against tissue autoantigens and cytokines [12, 13] denotes subtle deficits of the negative selection (delayed expression of MHC II pathway components), which may permit the development of autoimmune disorders upon lifelong exposure to environmental factors. Future research exploring how *AIRE* modulates the pathways involved in antigen processing and presentation may open novel ways for specific therapeutic interventions in autoimmune diseases linked to *AIRE* dysfunction.

## Author Contributions

A.C. Monteleone‐Cassiano performed the study, executed all the experiments, analysed and interpreted all the results and wrote the manuscript. P. Ferreirinha interpreted all the results and reviewed the manuscript. R.D. Pinto performed the experiments, interpreted all the results and reviewed the manuscript. U.G. Cipriano, A.F. Gembre and V.L.D. Bonato performed the experiment of T cell activation. GA Passos conceived the study, raised the hypothesis, interpreted all the results and reviewed the manuscript. N.L. Alves conceived the study, raised the hypothesis, interpreted all the results. E.A. Donadi conceived the study, raised the hypothesis, interpreted all the results, wrote and reviewed the manuscript. The authors read and approved the final manuscript.

## Funding

The following Brazilian research support agencies funded this work: Fundação de Amparo à Pesquisa do Estado de São Paulo (FAPESP, Grants 17/10780‐4 to G.A.P.; E.A.D. and 2019/23448‐3 to A.C.M.C.), Conselho Nacional de Desenvolvimento Científico e Tecnológico (CNPq, Grants 200236/2022‐9 to A.C.M.C., 305787/2017‐9 to G.A.P. and 302060/2019‐7 to E.A.D.), Coordenação de Aperfeiçoamento de Pessoal de Nível Superior (CAPES, Finance Code 001 and Grant 88887.695521/2022‐00 to A.C.M.C.) and INCTC/FAPESP #465539/2014‐9. The laboratory of N.L.A. is funded by “la Caixa” Foundation, under the project LCF/PR/HR23/52430019.

## Ethics Statement

Protocol no 003/2017‐1, entitled ‘Effect of *Aire* gene mutations (APSI Syndrome) induced by CRISpR‐Cas9 in protein conformation, transcriptome of mTEC cels and its interaction with thymocytes’, is in accordance with the Ethical Principles in Animal Research adopted by the National Council for Control of Animal Experimentation (CONCEA) and was approved in 03/28/2022 by the Local Animal Ethical Committee from the Ribeirão Preto Medical School of the University of São Paulo.

## Conflicts of Interest

The authors declare no conflicts of interest.

## Supporting information


**Figure S1:** Analysis of RNA sequencing data. (A) Heat‐map showing the large‐scale expression profiling of differentially expressed mRNAs involved in the antigen presentation pathway. Unsupervised heat‐maps and dendrograms were constructed using the R platform. Heat‐map legend: red = upregulated, orange = downregulated (Pearson's correlation metrics, fold change ≥ 1.5 and false discovery rate [FDR] < 0.05). (B) Genetic interaction network. A network of eight proteins identified by interaction with Aire, Ciita and INF‐γ. All nodes represent first order interaction. Coloured edges convey status of predicted network edge correspondingly cyan, curated database; magenta, experimentally determined; forest green, gene neighbourhood; red, gene fusion; navy blue, gene co‐occurrence; lawn green, text mining; black, co‐expression; lavender indigo, protein homology. Node colour signifies protein functionality. Additional nodes are considered based on prediction score ≥ 0.9 (for more details, refer to STRING database). (C) Expression profile of MHCII in mTECs. Flow cytometry analysis of expression of MHC II in mTECs *Aire* ^*WT*^ and *Aire* ^*−/−*^untreated or after 4 or 5 days of treatment with LPS (representative figure from three independent determinations). (D) Median fluorescence intensity was calculated by Prism GraphPad and represented in the graphic bar beside. Data shown (mean ± SD) are from three independent determinations and the significant difference between *Aire* ^*WT*^ and *Aire* ^*−/−*^ was analysed by the unpaired *t* test. The *Aire* ^*WT*^ cells are represented in black and the *Aire* ^*−/−*^ in red.
**Figure S2:** (A) FACS analysis of T‐cell proliferation (T‐cell assay) of DC alone + T cells in the first box and T cells alone in the second box. Used as positive control. (B) Median fluorescence intensity represented in the graphic bar. Data shown (mean ± SD) are from three independent determinations and the significant difference between *Aire* ^*WT*^ and *Aire* ^*−/−*^ was analysed by the unpaired *t* test. The *Aire* ^*WT*^ cells are represented in black and the *Aire* ^*−/−*^ in red. The figures are representative of at least three experiments.
**Figure S3:** (A) Gating strategy to confirm the phenotypic profile of CD69 expression in OT‐II lymphocytes. Identification of lymphocytes by FSC‐A/SSC‐A, exclusion of doublets by FSC‐H/FSC‐A, selection of live cells by labelling with fixable viability stain (FVS) probe, identification of lymphocytes by CD3/CD4 and CD69 expression, all with their respective fluorescence minus one (FMO) for analysis control. (B) T cell activation assay‐flow cytometry analysis for the CD69 marker after 18 h of culture. In first line, as negative control coculture with DCs and splenocytes without OVA^323–339^ peptide. As positive control, co‐culture with DCs and splenocytes in the presence of the OVA^323–339^ peptide (10 μg/mL). In the second line, co‐culture with mTEC *Aire* ^*WT*^ or mTEC *Aire* ^*−/−*^ and splenocytes without OVA^323–339^ peptide, stimulated or not with IFN‐γ (1 ng/μL). In the third line, co‐culture with mTEC *Aire* ^*WT*^ or mTEC *Aire* ^*−/−*^ and splenocytes without stimulated with IFN‐γ (1 ng/μL) in the presence of DCs. The figures are representative of at least four experiments. (C) Median fluorescence intensity in CD69‐positive fraction of the coculture without OVA^323–339^ peptide was calculated by Prism GraphPad and represented in the graphic bar beside. Data shown (mean ± SD) are from four independent determinations and the significant difference between *Aire* ^*WT*^ and *Aire* ^*−/−*^ was analysed by the unpaired *t* test.

## Data Availability

The raw mTEC RNA‐sequencing data used in this study were obtained from our previous publication [32] and are publicly available in the Gene Expression Omnibus (GEO) under accession number PRJNA763914. The data supporting the findings of this new study are included within the article and its Supporting Information.
