# Supplementary material for: The Role of the Autoimmune Regulator Gene in the Control of MHC II Antigen‐Processing and Presentation by Medullary Thymic Epithelial Cells
Source: HLA. 2025 Dec 22;106(6):e70516. doi: 10.1111/tan.70516 (PMC12722109; doi:10.1111/tan.70516)
Supplement: Supplementary file 1 — Figure S1: Analysis of RNA sequencing data. (A) Heat‐map showing the large‐scale expression profiling of differentially expressed mRNAs involved in the antigen presentation pathway. Unsupervised heat‐maps and dendrograms were constructed using the R platform. Heat‐map legend: red = upregulated, orange = downregulated (Pearson's correlation metrics, fold change ≥ 1.5 and false discovery rate [FDR] < 0.05). (B) Genetic interaction network. A network of eight proteins identified by interaction with Aire, Ciita and INF‐γ. All nodes represent first order interaction. Coloured edges convey status of predicted network edge correspondingly cyan, curated database; magenta, experimentally determined; forest green, gene neighbourhood; red, gene fusion; navy blue, gene co‐occurrence; lawn green, text mining; black, co‐expression; lavender indigo, protein homology. Node colour signifies protein functionality. Additional nodes are considered based on prediction score ≥ 0.9 (for more details, refer to STRING database). (C) Expression profile of MHCII in mTECs. Flow cytometry analysis of expression of MHC II in mTECs Aire WT and Aire −/− untreated or after 4 or 5 days of treatment with LPS (representative figure from three independent determinations). (D) Median fluorescence intensity was calculated by Prism GraphPad and represented in the graphic bar beside. Data shown (mean ± SD) are from three independent determinations and the significant difference between Aire WT and Aire −/− was analysed by the unpaired t test. The Aire WT cells are represented in black and the Aire −/− in red. Figure S2: (A) FACS analysis of T‐cell proliferation (T‐cell assay) of DC alone + T cells in the first box and T cells alone in the second box. Used as positive control. (B) Median fluorescence intensity represented in the graphic bar. Data shown (mean ± SD) are from three independent determinations and the significant difference between Aire WT and Aire −/− was analysed by the unpaired t test. The Ai [file TAN-106-e70516-s001.docx]

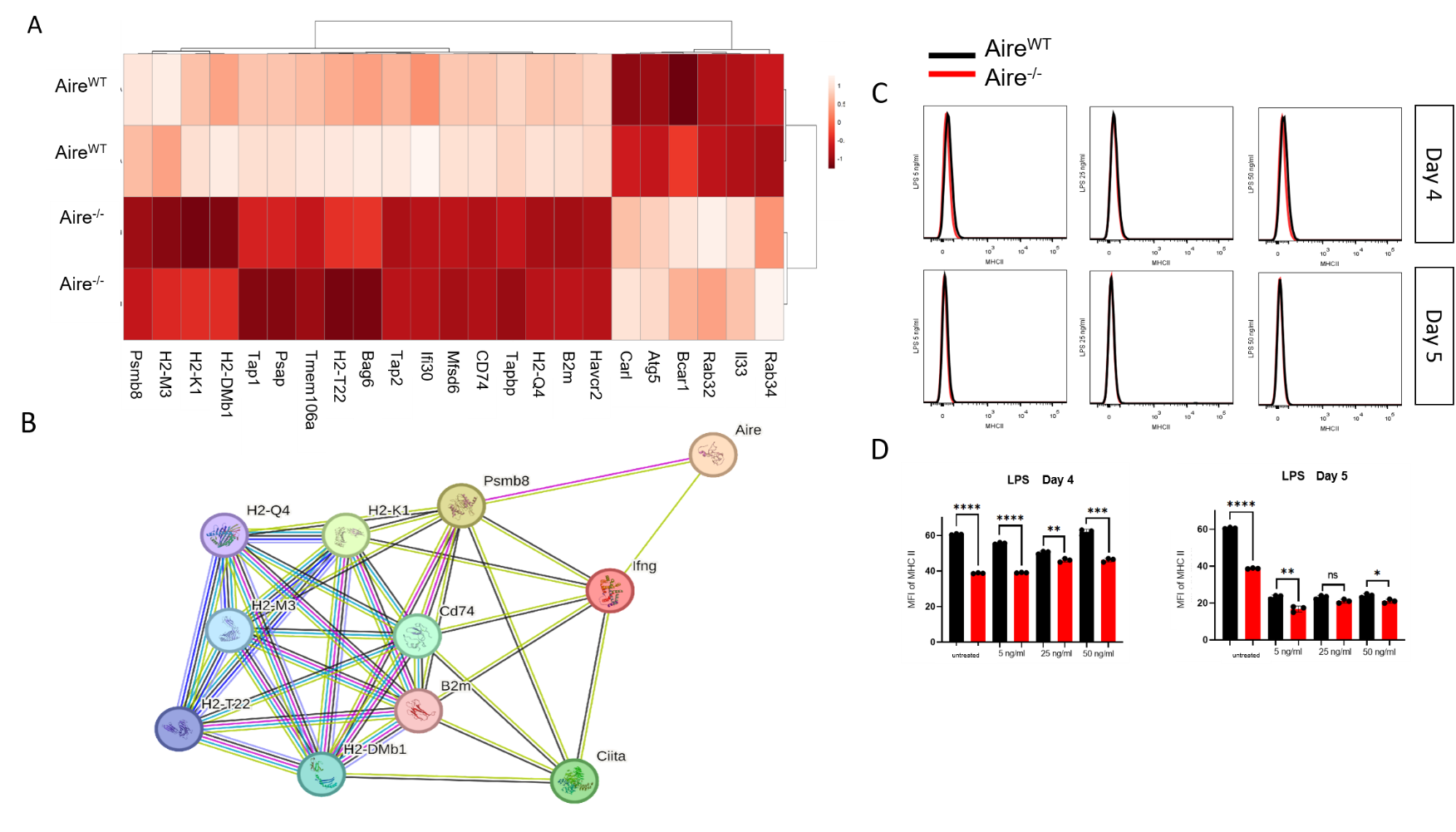


**Supplementary Figure 1** Analysis of RNA sequencing data. **A‑** Heat‑map showing the large‑scale expression profiling of differentially expressed mRNAs involved in the antigen presentation pathway. Unsupervised heat‑maps and dendrograms were constructed using the R platform. Heat‑map legend: red=upregulated, orange =downregulated (Pearson’s correlation metrics, fold change ≥1.5 and false discovery rate (FDR) < 0.05). **B-** Genetic Interaction network. A network of 8 proteins identified by interaction with Aire, Ciita and INF-γ. All nodes represent first order interaction. Colored edges convey status of predicted network edge correspondingly cyan, curated database; magenta, experimentally determined; forest green, gene neighborhood; red, gene fusion; navy blue, gene co‑occurrence; lawn green, text mining; black, co‑expression; lavender indigo, protein homology. Node color signifies protein functionality. Additional nodes are considered based on prediction score≥0.9 (for more details, refer to STRING database). **C**- Expression profile of MHCII in mTECs. Flow cytometry analysis of expression of MHC II in mTECs *Aire^WT^* and *Aire^-/-^*untreated or after 4 or 5 days of treatment with LPS (representative figure from three independent determinations). **D-** Median fluorescence intensity was calculated by Prism GraphPad and represented in the graphic bar beside. Data shown (mean±SD) are from three independent determinations, and the significant difference between *Aire^WT^* and *Aire^-/-^* was analyzed by the unpaired t‑test. The *Aire^WT^* cells are represented in black and the *Aire^-/-^* in red.

**
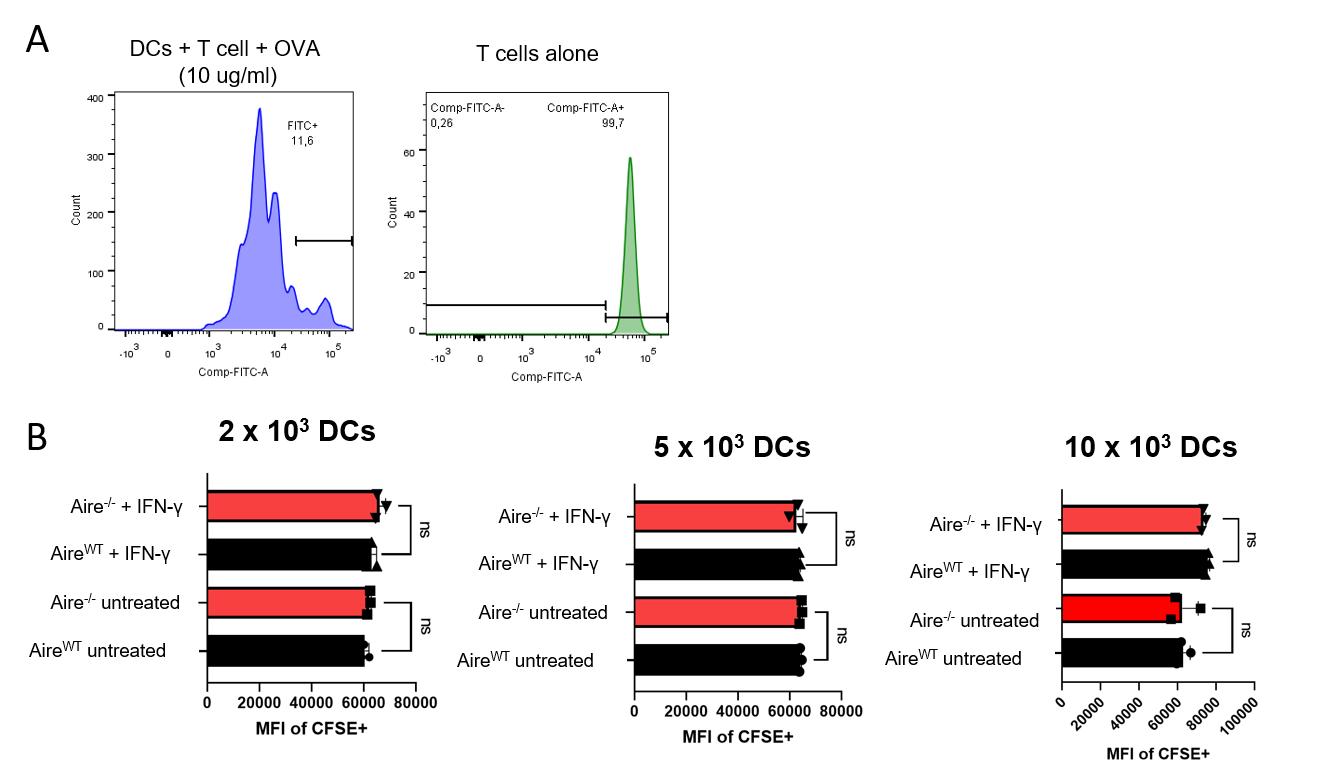
**

**Supplementary Figure 2- A-** FACS analysis of T-cell proliferation (T-cell assay) of DC alone + T cells in the first box, and T cells alone in the second box. Used as positive control. **B-** Median fluorescence intensity represented in the graphic bar. Data shown (mean±SD) are from three independent determinations, and the significant difference between *Aire^WT^* and *Aire^-/-^* was analyzed by the unpaired t‑test. The *Aire^WT^* cells are represented in black and the *Aire^-/-^* in red. The figures are representative of at least three experiments.

**
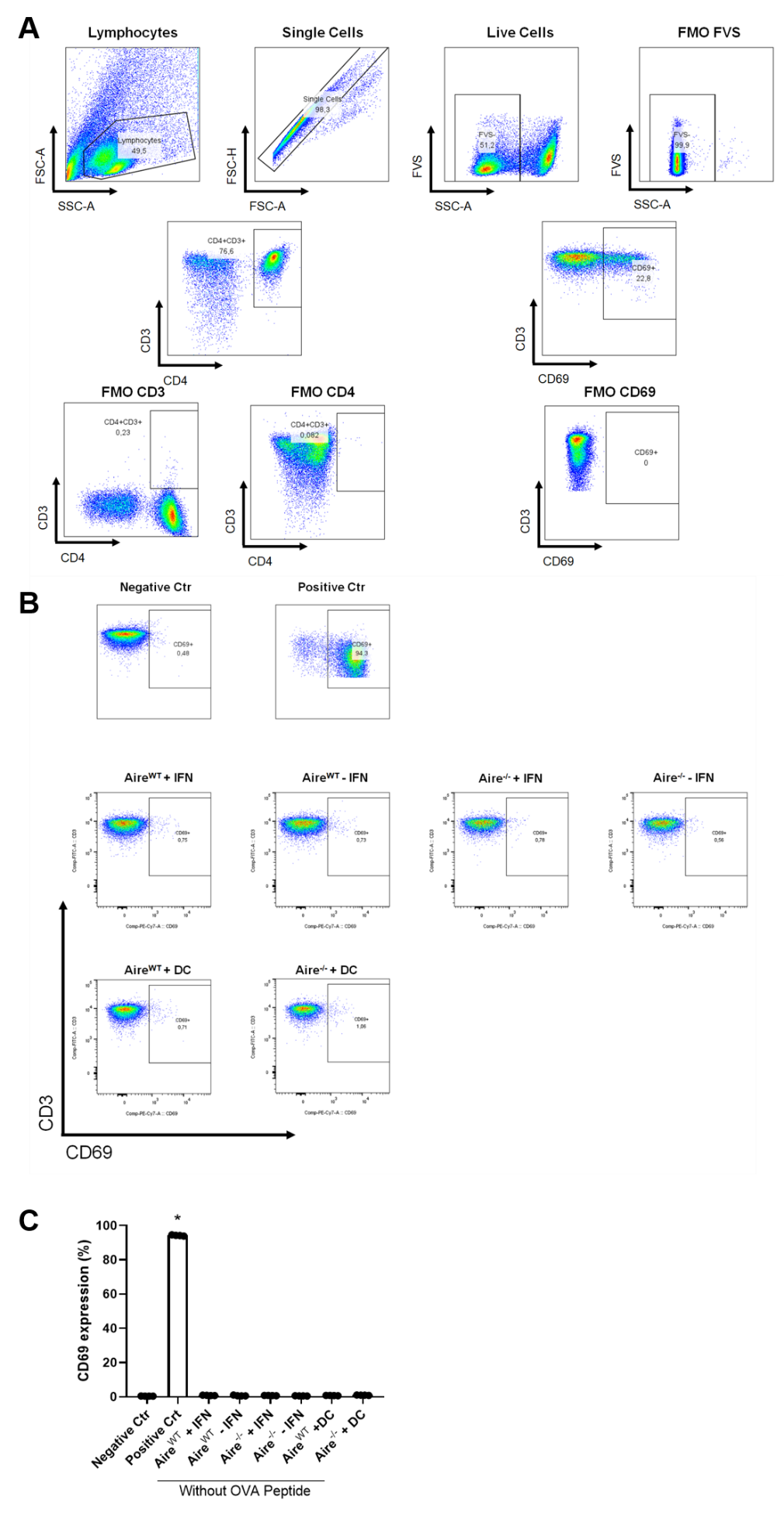
**

**Supplementary Figure 3- A-** Gating strategy to confirm the phenotypic profile of CD69 expression in OT-II lymphocytes. Identification of lymphocytes by FSC-A/SSC-A, exclusion of doublets by FSC-H/FSC-A, selection of live cells by labeling with Fixable Viability Stain (FVS) probe, identification of lymphocytes by CD3/CD4 and CD69 expression, all with their respective fluorescence minus one (FMO) for analysis control. **B-** T cell activation assay- flow cytometry analysis for the CD69 marker after 18 hours of culture. In first line, as negative control coculture with DCs and splenocytes without OVA^323-339^ peptide. As positive control, coculture with DCs and splenocytes in the presence of the OVA^323-339^ peptide (10μg/ml). In In the second line, coculture with mTEC *Aire^WT^* or mTEC *Aire ^-/-^* and splenocytes without OVA^323-339^ peptide, stimulated or not with IFN- γ (1 ng/μl). In the third line, coculture with mTEC *Aire^WT^* or mTEC *Aire ^-/-^* and splenocytes without stimulated with IFN- γ (1 ng/μl) in the presence of DCs. The figures are representative of at least four experiments. **C-** Median fluorescence intensity in CD69 positive fraction of the coculture without OVA^323-339^ peptide was calculated by Prism GraphPad and represented in the graphic bar beside. Data shown (mean±SD) are from four independent determinations, and the significant difference between *Aire^WT^* and *Aire^-/-^* was analyzed by the unpaired t‑test.
